# Effects of acute exercise fatigue on the spatiotemporal dynamics of resting-state large-scale brain networks

**DOI:** 10.3389/fnins.2023.986368

**Published:** 2023-01-20

**Authors:** Shanguang Zhao, Hao Lin, Aiping Chi, Yuanyuan Gao

**Affiliations:** ^1^Institute of Physical Education, Shaanxi Normal University, Xi’an, China; ^2^Faculty of Sports and Exercise Science, Universiti Malaya, Kuala Lumpur, Malaysia

**Keywords:** acute exercise fatigue, resting-state EEG, microstates, large-scale brain networks, energy

## Abstract

**Introduction:**

Various approaches have been used to explore different aspects of the regulation of brain activity by acute exercise, but few studies have been conducted on the effects of acute exercise fatigue on large-scale brain functional networks. Therefore, the present study aimed to explore the effects of acute exercise fatigue on resting-state electroencephalogram (EEG) microstates and large-scale brain network rhythm energy.

**Methods:**

The Bruce protocol was used as the experimental exercise model with a self-controlled experimental design. Thirty males performed incremental load exercise tests on treadmill until exhaustion. EEG signal acquisition was completed before and after exercise. EEG microstates and resting-state cortical rhythm techniques were used to analyze the EEG signal.

**Results:**

The microstate results showed that the duration, occurrence, and contribution of Microstate C were significantly higher after exhaustive exercise (*p’s* < 0.01). There was a significantly lower contribution of Microstate D (*p* < 0.05), a significant increase in transition probabilities between Microstate A and C (*p* < 0.05), and a significant decrease in transition probabilities between Microstate B and D (*p* < 0.05). The results of EEG rhythm energy on the large-scale brain network showed that the energy in the high-frequency β band was significantly higher in the visual network (*p* < 0.05).

**Discussion:**

Our results suggest that frequently Microstate C associated with the convexity network are important for the organism to respond to internal and external information stimuli and thus regulate motor behavior in time to protect organism integrity. The decreases in Microstate D parameters, associated with the attentional network, are an important neural mechanism explaining the decrease in attention-related cognitive or behavioral performance due to acute exercise fatigue. The high energy in the high-frequency β band on the visual network can be explained in the sense of the neural efficiency hypothesis, which indicates a decrease in neural efficiency.

## 1. Introduction

Fatigue causes a decrease in cognitive ability and physical performance (Casanova et al., 2013; Alder et al., 2019, 2021; Coco et al., 2020). Fatigue during prolonged strenuous exercise is affected by a variety of central nervous systems (Proschinger and Freese, 2019). The collaborative working of multiple brain regions forms a distinct network structure, reflecting the relationship between cognitive ability and the brain (van den Heuvel and Hulshoff Pol, 2010). Insights into the features of the large-scale brain networks underlying fatigue provides new perspectives to unravel the neural basis of central fatigue.

Regarding the effect of acute exercise fatigue on a resting-state EEG, power changes in alpha and beta waves in frontal and parietal regions have been widely reported (Moraes et al., 2007; Schneider et al., 2009; Brummer et al., 2011; Enders et al., 2016). However, the current study is insufficient to determine the modulation of EEG rhythms on specific brain regions by acute exercise fatigue, as this varies with exercise intensity, pattern, and subject exercise preference (Schneider et al., 2009; Brummer et al., 2011). Therefore, it is questionable to explain the effects of acute motor fatigue on related cognitive functions by power changes in EEG rhythms on specific brain regions. However, it is worth noting that different EEG rhythms can exist within the same neural network or interact within different neural networks, and that different brain activities can cause competition between different EEG rhythms within the same neural network (Steriade, 2001; Varela et al., 2001; Mantini et al., 2007), but there are no studies on the effect of exercise on the energy of EEG rhythms within neural networks. Therefore, to more accurately explain the neural mechanisms underlying the effects of acute motor fatigue on specific cognitive functions, we applied a new method, REsting-state COrtex Rhythms (RECOR), which allows for the detailed localization of large-scale network cortical sources of resting-state EEG rhythms. The RECOR toolkit not only reconstructs the cortical current density of EEG rhythms, but also calculates the energy of each rhythm over eight large-scale brain networks, including visual, sensorimotor, dorsal attention, ventral attention, limbic system, frontoparietal, default mode, and deep structural networks.

However, maintaining cognitive activity requires not only the intensity of activation of brain networks, but also the duration of activation. EEG microstates are a reliable method for analyzing brain network dynamics, which allows for studying the dynamic pattern changes of individuals in large-scale brain networks under different conditions on millisecond time scales. EEG microstates are of great relevance to examining various diseases and behavioral state changes in humans, but there are few studies on the effect of exercise on microstates. However, it is interesting to note that the four typical microstates (Microstate A, B, C, and D) obtained from previous studies correspond to the speech processing network, visual network, convexity network, and attention network, respectively (Lehmann et al., 1998; Damoiseaux et al., 2006; Seeley et al., 2007; Britz et al., 2010). Among them, the projection network, which mainly includes the anterior insula and anterior cingulate cortex, has the function of detecting, receiving, and integrating internal and external information stimuli, and subsequently switching to the relevant processing systems (Seeley et al., 2007; Menon, 2011; Chiong et al., 2013; Uddin, 2015). Moreover, previous studies have shown that acute exercise fatigue leads to enhanced activation of the insula cortex (Hilty et al., 2011a). Therefore, we hypothesized that the drastic physiological changes or alterations in muscle properties induced by fatiguing exercise tasks would be afferent to insula regions, which in turn would cause changes in Microstate C, associated with the convexity network. Our study is also focused on Microstate B, associated with the visual network, and Microstate D, associated with the attention network, since these two networks are involved in various cognitive activities (e.g., attention, anticipation, and decision making) of athletes during the competition (Wright et al., 2010, 2013). Thus, the activity of Microstate B and D may be an important neural mechanism for understanding changes in various cognitive activities in athletes after acute exercise fatigue.

Both activation and switching of brain networks affect relevant cognitive performance, yet previous studies have not explored the neural basis of fatigue in these two dimensions. To address the shortcomings of previous studies, this study collected EEG signals from 30 male college students majoring in physical education before and after exercise by setting up a Bruce exercise protocol through atreadmill, and used EEG microstate and cortical rhythm techniques to explore the effects of acute exercise fatigue on the temporal dynamics and rhythmic energy of large-scale brain networks, which in turn revealed their effects on the athletes’ general cognitive performance or specific cognitive performance. In the present study, we hypothesized that acute motor fatigue would alter the functional state of certain resting-state large-scale brain networks, as reflected mainly by microstate indicators and changes in EEG rhythm energy on large-scale brain networks.

## 2. Participants and methods

### 2.1. Participants

Thirty male college students majoring in physical education volunteered to participate in this experiment. All participants had normal or corrected visual acuity and were right-handed; had no musculoskeletal, cardiovascular, psychiatric, or neurological diseases or medical history. In the 24 h prior to the test, they did no strenuous exercise, took no medication, did not smoke, consumed no alcohol or coffee, or experienced no mood swings. The procedure of the experiment was clarified to all participants, and the participants read and signed the written informed consent form before testing. The experiment was approved by the Academic Ethics Committee of Shaanxi Normal University and was in accordance with the Declaration of Helsinki. Demographic information is shown in Table 1.

**TABLE 1:** Subject demographic characteristics.

Test index	Results
Age (years)	21.97 ± 2.14
Height (cm)	178 ± 5.33
Weight (kg)	70.21 ± 8.37
BMI (kg/m^2^)	22.02 ± 2.14
Skeletal muscle (%)	39.48 ± 3.72
Body fat (%)	13.92 ± 4.63
Basal metabolism/d (Kcal)	1,883 ± 187.2
Training time (years)	3.52 ± 0.45

### 2.2. Exercise protocol

Using the Bruce exercise protocol, participants wore a Polar and portable blood pressure monitor on a treadmill (h/p/cosmos cos10253 Germany). The treadmill started from the first level of 2.7 km/h and 10% slope, and the speed and slope increased every 3 min. The detailed parameters of each level are shown in Table 2. During the experiment, a recorder was present at all times to monitor participant’s ambulatory heart rate and blood pressure during exercise. The Rating of Perceived Exertion (RPE) was used to evaluate the subjective feeling of the participants at the end of each exercise level.

**TABLE 2:** Bruce program levels of exercise load.

Parameters	Level 1	Level 2	Level 3	Level 4	Level 5	Level 6	Level 7
Speed (km/h)	2.7	4.0	5.4	6.7	8.0	8.8	9.6
Slope (%)	10	12	14	16	18	20	22
Duration (min)	3	3	3	3	3	3	3

The exercise was terminated when the participants experienced any three of the following four conditions: (1) participants exhibited respiratory distress; (2) participants had a systolic blood pressure greater than 150 mm Hg and a diastolic blood pressure greater than 75 mm Hg; (3) the participant’s heart rate approached or reached his or her maximum heart rate (HRmax = 208-0.7*age) (Tanaka et al., 2001); (4) participants had RPE values of 18–19 and were unable to continue exercise even after encouragement.

### 2.3. EEG acquisition and pre-processing

The EEG data of the participants were recorded for 5 min immediately after the termination of exercise. The first 3 min of EEG data were selected for analysis (Carroll et al., 2017). A high-resolution EEG acquisition system (Neuroscan, USA) with 32 conductive polar caps extended by the International 10–10 system was used to record the EEG signals. Online EEG data were recorded using 0.05–100 Hz band-pass filtering with a sampling frequency of 1,000 Hz/conductor, using bilateral mastoids as reference electrodes and forehead grounding. Vertical electrooculography (EOG) activity was recorded with electrodes placed above and below the left eye, and horizontal EOG activity was recorded with electrodes placed laterally at both eyes. The impedance between all electrodes and the scalp was less than 10 kΩ.

Raw EEG data was pre-processed using EEGLAB (Version R2013b, San Diego, CA, USA), an open-source toolbox running on MATLAB environment (Version R201 3b, MathWorks, United States). A 0.5–45 Hz bandpass filter, as well as a 50 Hz notch filter, were applied to the EEG data using a finite impulse response filter. Thereafter, the EEG waveforms were epoched into segments of 2-s duration and remontaged to an average reference. An independent component analysis (ICA) procedure was used to identify and extract artifact components, and remove the segment of the sources containing eye blink artifacts, eye movement, and EMG artifacts (High-frequency signals).

### 2.4. Cortex rhythms (RECOR)

REsting-state COrtex Rhythms ^1^ was used to estimate the EEG rhythms’ power in the eight large-scale brain networks (Lei et al., 2011; Lei, 2012). The EEG forward model was restricted to a high-density canonical cortical mesh extracted from a structural MRI of a neurotypical male in Fieldtrip software.^2^ The mesh had 8,196 vertices uniformly distributed on the gray-white matter interface. Each vertex node was assumed to have one dipole oriented perpendicularly to the surface. The 32 electrodes were registered to the scalp surface, and the lead-field matrix (32 × 8,196) was calculated within a three-shell spherical head model, including the scalp, skull, and brain.

REsting-state COrtex Rhythms included two steps calculating the power of EEG rhythms in each brain network. Firstly, network-based source imaging (NESOI) was employed to estimate the cortical sources of EEG rhythms (Lei et al., 2011). Eight large-scale brain networks were used as the covariance priors of the EEG source reconstruction using parametric empirical Bayesian. Seven large-scale networks were identified based on 1,000 resting-state functional connectivity: visual, somatomotor, dorsal attention, ventral attention, limbic, frontoparietal, and default networks (Yeo et al., 2011). Considering the importance of the deep brain structure (thalamus and striatum), we used the anatomical mask of the WFU pick atlas to construct the eighth large-scale networks (Maldjian et al., 2003). The 8,196 vertices were separated into eight subsets based on their nearest neighbor voxel in the large-scale brain network templates. The covariance prior Vi is from the ith brain network and is an 8,196 × 8,196 covariance basis matrix, the columns and rows of which were assigned with the Green function of the mesh adjacency matrix if their corresponding vertices were involved in the network, and the other terms were zero (Lei et al., 2011). The intensity of the neuroelectric sources of EEG rhythms was iteratively estimated by the restricted maximum likelihood (ReML) algorithm. It has been shown that NESOI is quite efficient when compared to other inverse methods, such as the weighted minimum norm solution, low-resolution brain electromagnetic tomography (LORETA), and the multiple sparse prior model (MSP) (Lei et al., 2011).

The second step of RECOR is averaging the solutions of NESOI across all vertices of a given large-scale brain network. Rather than estimating the punctual EEG source patterns of each rhythm, RECOR focused on the large-scale distribution of the EEG source and calculated an averaged current density at each network. This is in line with the low spatial resolution of the adopted technique. As the number of electrodes (32) is much lower than that of the unknown current density at each vertex (8,196), solutions to the EEG inverse problem are under-determined and ill- conditioned. This averaging step may minimize the effects of poor NESOI estimates in the deep brain structure at which the estimation of EEG sources could be imprecise, especially using an EEG spatial sampling from 32 electrodes (10–10 system). In summary, the RECOR software reported the current density for all of the vertices and the eight brain networks.

### 2.5. Resting EEG microstates

We used Cartool 3.70 software ^3^ to perform separate microstate analyses of EEG before and after exercise (Brunet et al., 2011). The steps are as follows: First, band-pass filtering of 2–20 Hz was performed, and the bilateral mastoid (M1, M2) reference was converted to a whole-brain average reference (Michel and Koenig, 2018). Second, the instantaneous topography at the local peak of GFP was selected for k-means clustering, and a meta-criterion containing seven independent criteria was applied to determine the number of optimal clusters for each subject (Brechet et al., 2019). The polarity of topographic maps was ignored for both clustering processes. Finally, the “original map”, that is the topographic map at the peak of GFP of each subject was assigned to the population microstate map with the greatest spatial correlation, thus obtaining the microstate sequence of each subject, and the polarity of the topographic map was again ignored in this process.

For each subject, the following microstate parameters were calculated: (1) global explained variance, the percentage of total variance explained by a given microstate; (2) duration, the average duration for which a microstate category remains in a relatively stable state; (3) the frequency of occurrence, the average number of occurrences per second of a microstate category; (4) contribution, the total duration of a microstate category as a percentage of the total resting-state EEG time; and (5) transition probability, the ratio of the number of switches from one microstate to another to the total number of switches between all microstates.

### 2.6. Statistical analysis

Statistical analysis was performed using IBM SPSS 26.0, and the Shapiro–Wilk test showed that all groups of data obeyed a normal distribution. A 2 (time: before and after exhaustive exercise) × 8 (large-scale brain networks: visual network, sensorimotor network, dorsal attention network, ventral attention network, limbic system network, frontoparietal network, default mode network, and deep brain structure network) two-factor repeated measures ANOVA was used to compare the differences in EEG rhythm energy on eight large-scale brain networks before and after exhaustive exercise. A 2 (time: before and after exhaustive exercise) × 4 (microstate categories: A, B, C, D) two-factor repeated-measures ANOVA was used to compare the changes in the temporal parameters of each microstate (mean duration, frequency of occurrence, and time coverage) before and after force exhaustion exercise. A 2 (time: before and after exhaustive exercise) × 12 (pairs: A→B, B→A, A→C, C→A, A→D, D→A, B→C, C→B, B→D, D→B, C→D, D→C) two-factor repeated measures ANOVA was used to compare the differences in transition probabilities between each microstate before and after force exhaustion exercise. All repeated measures ANOVAs were analyzed using Mauchly’s spherical hypothesis test to determine whether the spherical hypothesis was met, and those that did not meet the spherical hypothesis were corrected using the Greenhouse–Geisser method. *Post hoc* pairwise comparisons were corrected using the Bonferroni method.

## 3. Results

### 3.1. Behavioral and physiological parameters

The behavioral and physiological parameters of the participants at the end of the exercise are shown in Table 3. According to the exhaustion criteria, all participants felt dyspnea at the end of exercise; their heart rates were at or near their maximum heart rates; their mean systolic and diastolic blood pressures were greater than 150 and 75 mm Hg; their RPE levels were greater than 18, indicating that all participants were in a state of exhaustion.

**TABLE 3:** Behavior and physiological parameters of participants at the end of exercise.

Variable	Mean ± SD
HRmax (bpm)	189.47 ± 12.35
Systolic blood pressure (mm Hg)	156.47 ± 18.56
Diastolic blood pressure (mm Hg)	77.56 ± 14.12
Rating of Perceived Exertion	19.24 ± 1.15
Duration of exercise (min)	19.20 ± 3.07

### 3.2. EEG microstate analysis

**FIGURE 1:**
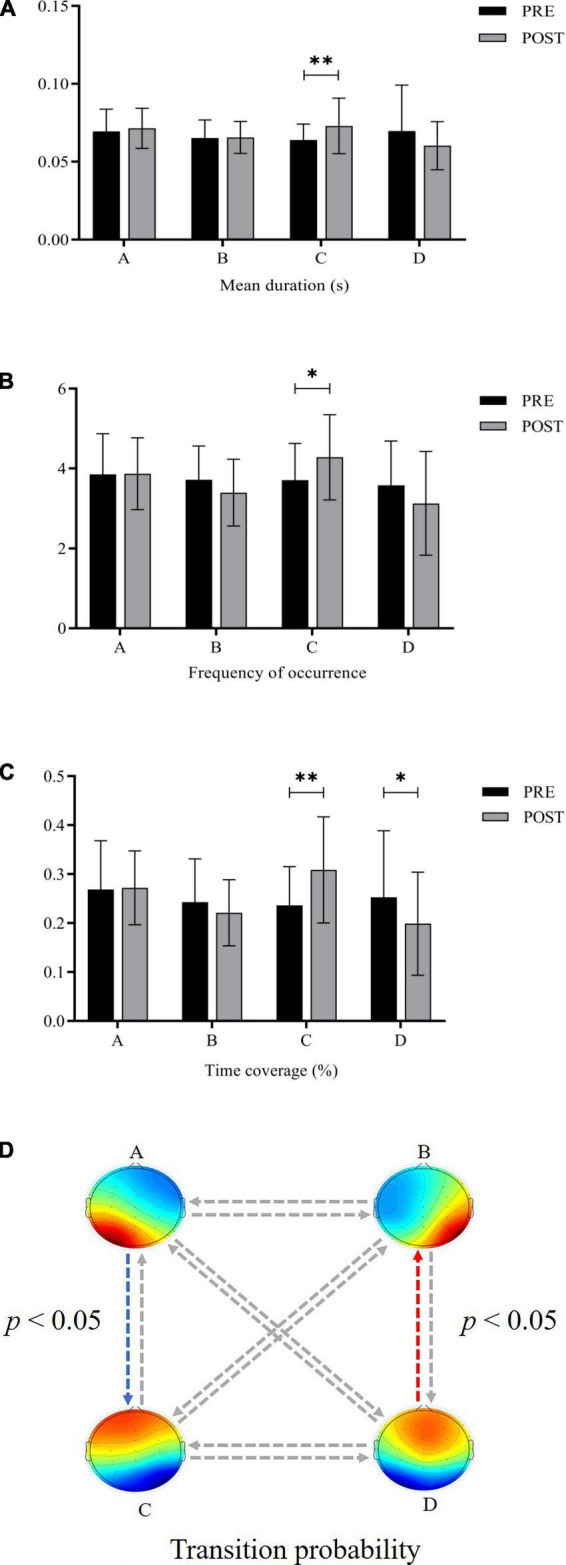
Comparison of microstate parameters before and after exhaustive exercise **(A)** duration **(B)** occurrence **(C)** time coverage **(D)** transition probability (*: *p* < 0.05; ^**^: *p* < 0.01; blue arrow: increase; red arrow: decrease).

For microstate duration, the ANOVA found that the main effect of time was not significant, *F*(1,19) = 0.229, *p* > 0.05, ${\text{η}}_{2}^{\text{p}}$ = 0.012, the main effect of microstate class was not significant, *F*(3,57) = 0.712, *p* > 0.05, ${\text{η}}_{2}^{\text{p}}$ = 0.036, and the interaction effect was significant, *F*(3,57) = 3.264, *p* < 0.05, ${\text{η}}_{2}^{\text{p}}$ = 0.147. *Post-hoc* tests found that the durations of Microstate C increased significantly after exhaustive exercise (*p* < 0.01) (Figure 1A).

The ANOVA on the microstate occurrence yielded a significant interaction effect, *F*(3,57) = 3.390, *p* < 0.05, ${\text{η}}_{2}^{\text{p}}$ = 0.151. *Post-hoc* tests revealed that Microstate C was significantly more frequent after exhaustive exercise (*p* < 0.05) (Figure 1B). There were no significant main effects.

For microstate time coverage, the main effect of time was not significant, *F*(1,19) = 0.388, *p* > 0.05, ${\text{η}}_{2}^{\text{p}}$ = 0.020, the main effect of the microstate class was not significant, *F*(3,57) = 1.356, *p* > 0.05, ${\text{η}}_{2}^{\text{p}}$ = 0.067, and the interaction between these two factors was significant, *F*(3,57) = 3.963, *p* < 0.05, ${\text{η}}_{2}^{\text{p}}$ = 0.173. *Post-hoc* tests demonstrated that the time coverage of Microstate C increased significantly (*p* < 0.01) and that of Microstate D decreased significantly (*p* < 0.05) after exhaustive exercise (Figure 1C).

Concerning the transition probability, the 2 (time) × 12 (pairs) ANOVA showed a significant interaction [*F*(3.287,62.462) = 2.929, *p* < 0.05, ${\text{η}}_{2}^{\text{p}}$ = 0.134] with the observed transition percentage. Follow-up t-tests indicated that the transition probability between Microstate A and C increased significantly after exhaustive exercise (*p* < 0.05), while the transition probability between Microstate B and D decreased significantly (*p* < 0.05) (Figure 1D).

### 3.3. Large-scale brain network energy analysis

The results for rhythmic energy on large-scale brain networks (Tables 4, 5) showed that none of the main effects of time was significant (*p* > 0.05), that all of the main effects for brain networks were significant (*p* < 0.001), and none of the interactions between time and brain networks was significant (*p* > 0.05). Further paired-samples *t*-test analysis showed that the energy of the high-frequency β band was significantly higher on the visual network after exhaustive exercise.

**TABLE 4:** Rhythmic energy values on large-scale brain networks before and after exhaustive exercise.

		δ	θ	α 1	α 2	β 1	β 2	γ
Visual network	PRE	3.9926 ± 1.8808	3.8724 ± 1.2666	4.6480 ± 2.1547	4.3175 ± 1.8391	3.6491 ± 1.7762	**2.5673 ± 1.3426**	3.0190 ± 1.8495
	POST	4.0735 ± 1.8311	4.2048 ± 1.6988	4.4887 ± 1.9956	5.1135 ± 2.1277	4.2687 ± 1.9004	**3.5223 ± 1.7223**	3.3356 ± 1.7346
Sensorimotor network	PRE	4.1339 ± 1.5138	3.9540 ± 1.0485	4.1195 ± 1.1696	3.4633 ± 1.3029	3.0056 ± 1.1886	2.5429 ± 1.3329	2.9563 ± 2.2665
	POST	3.7570 ± 1.0218	4.3616 ± 1.5614	4.3766 ± 2.2192	4.4110 ± 2.7453	3.8013 ± 2.0699	3.2510 ± 1.8643	2.9791 ± 1.1853
Dorsal attention network	PRE	4.4508 ± 2.1188	4.0971 ± 1.1795	4.3956 ± 1.5643	4.1536 ± 1.6631	3.6307 ± 1.7569	2.8068 ± 1.6085	3.1444 ± 2.3217
	POST	3.8934 ± 1.1765	4.4984 ± 1.8278	4.3788 ± 2.2300	5.1060 ± 3.0781	4.2811 ± 2.3044	3.6482 ± 2.0030	3.1201 ± 1.2000
Ventral attention network	PRE	4.6401 ± 2.0858	4.1946 ± 1.0745	4.1012 ± 1.1394	3.6887 ± 1.6351	3.2724 ± 1.3343	2.7660 ± 1.6565	3.3486 ± 2.3782
	POST	4.1438 ± 1.7393	4.4548 ± 2.2902	4.4543 ± 2.8569	4.2706 ± 3.2190	3.8570 ± 2.3931	3.3628 ± 2.1847	3.3627 ± 1.3899
Limbic system network	PRE	4.7820 ± 2.0429	4.3605 ± 1.3014	4.5111 ± 1.4596	4.0595 ± 1.9937	3.4912 ± 1.4765	2.7753 ± 1.5001	3.5014 ± 2.1262
	POST	4.7946 ± 2.0180	4.8930 ± 2.2044	5.0671 ± 3.0424	4.8921 ± 3.2895	4.3722 ± 2.4622	3.6607 ± 2.3393	3.7531 ± 1.4082
Frontoparietal network	PRE	6.2691 ± 3.5351	5.2077 ± 1.7237	4.8272 ± 2.1404	4.2226 ± 1.9815	3.8803 ± 1.8939	3.4227 ± 1.8283	4.3003 ± 2.7464
	POST	5.4529 ± 2.8468	5.3942 ± 2.9467	5.0528 ± 3.1207	5.0867 ± 3.3187	4.2954 ± 2.1974	4.5139 ± 3.2215	4.4193 ± 1.9868
Default mode network	PRE	5.7403 ± 3.2470	5.0313 ± 1.6216	4.9157 ± 1.9813	4.6786 ± 2.1210	4.0315 ± 1.8628	3.2949 ± 1.9590	4.2091 ± 2.7649
	POST	5.1832 ± 2.4752	5.1542 ± 2.7158	5.2515 ± 3.3028	5.5050 ± 3.6218	4.6596 ± 2.5975	4.3760 ± 2.7973	4.4279 ± 1.9020
Deep structural network	PRE	2.4830 ± 0.9499	2.4049 ± 0.6311	2.3194 ± 0.7552	2.1301 ± 1.2288	1.8999 ± 0.6572	1.5824 ± 0.8557	1.7362 ± 1.0036
	POST	2.1992 ± 0.9630	2.3511 ± 1.0912	2.1790 ± 1.1936	2.2055 ± 1.4048	2.1128 ± 1.2171	1.6751 ± 0.9562	1.6206 ± 0.7175

Bold values indicate statistical significance (*p* < 0.05).

**TABLE 5:** Results of two-factor ANOVA for brain network energy values.

Frequency bands	The master effect of time	The main effect of brain networks	The interaction of conditions with brain networks
δ	*F*(1,19) = 0.366	*F*(2.202,41.836) = 31.458***	*F*(2.168,41.197) = 0.866
θ	*F*(1,19) = 0.306	*F*(2.383,45.270) = 25.079***	*F*(2.753,52.306) = 0.507
α1	*F*(1,19) = 0.078	*F*(2.494,47.386) = 24.887***	*F*(2.962,56.273) = 0.787
α2	*F*(1,19) = 1.265	*F*(2.812,53.432) = 27.779***	*F*(2.818,53.537) = 1.048
β1	*F*(1,19) = 1.315	*F*(3.572,67.860) = 26.050***	*F*(3.022,57.421) = 0.884
β2	*F*(1,19) = 2.156	*F*(2.673,50.791) = 23.058***	*F*(2.097,39.835) = 1.556
γ	*F*(1,18) = 0.036	*F*(2.903,52.258) = 26.446***	*F*(2.819,50.734) = 0.245

***p < 0.001.

## 4. Discussion

This is the first study to explore the effects of acute motor fatigue on resting-state EEG microstates and large-scale brain network energy. We used the Bruce protocol as an experimental model for exercise. All participants were in a state of exhaustion at the end of exercise (Dyspnea, RPE values greater than 18, heart rate at or near my maximum heart rate, and systolic and diastolic blood pressures greater than 150 and 75 mm Hg), identified as acute exercise fatigue production. Consistent with our hypothesis, participants experienced significant changes in certain microstate category parameters and large-scale brain network energy after exhaustive exercise, which provides new ideas for investigating the neural mechanisms by which locomotor fatigue affects cognitive or behavioral performances.

### 4.1. EEG microstate analysis

The study found a significant increase in the duration, occurrences, and contribution of microstate C after motion, consistent with previous studies (Spring et al., 2017, 2018). Microstate C is associated with the convex network, including the anterior cingulate cortex bilaterally in the inferior frontal gyrus, and the right anterior insula. The anterior insula is sensitive to changes in physiological signals such as blood pressure, heart rate, and respiration, constituting an important structure for processing sensory stimuli and transmitting physiological signals (Seeley et al., 2007; Britz et al., 2010). The changes in blood pressure and heart rate induced by exhaustive exercise may be transmitted to the anterior insula region, which may cause changes in microstate C parameters. Hilty et al. (2011a) revealed enhanced activity of the anterior insula exercise cessation in a muscle fatigue experiment using a grip strength device on participants with right-hand finger flexion and extension. Subsequently, Hilty et al. (2011b) found enhanced communication between the middle and anterior insula and sensorimotor cortex immediately after exercise in an exertional cycling task. Therefore, we hypothesize that the elevation of Microstate C after exhaustive exercise may cause enhanced connectivity between the convexity network and the sensorimotor network, which is crucial for the brain to respond to changes in physiological signals and thus regulate motor behavior in time to protect organismal integrity.

Second, we found that contribution of Microstate D was significantly lower after exhaustive exercise. Microstate D correlates with activity in the right dorsal and ventral regions of the frontal and parietal lobes, corresponding to the attentional network (Britz et al., 2010). Studies have found reduced brain activation in the frontal cortex (Mekari et al., 2015; Bao et al., 2019) and the reduced efficiency of attention-related networks (Schmitt et al., 2019; Buchel et al., 2021) due to acute high-intensity exercise support the present study on the modulation of Microstate D contribution. In addition, the relationship between exercise intensity and cognitive or exercise performance appears to be an inverted “U” shape; beneficial effects occur at low and moderate-intensity exercise but deteriorate at high-intensity exercise (McMorris et al., 2011, 2015; Alves et al., 2014).

In the field of sports competition, the ability to multi-target tracking is critical for athletic performance in team sports (basketball, football, and hockey), because players need to pay attention not only to ball transitions but also to changes in the positions of opponents and teammates during the game, and even they need to judge the intentions of teammates and opponents based on their body movements, eyes, and expressions (Memmert and Perl, 2009). However, the multi-target tracking process mainly involves the enhanced activation of the dorsal attention network (Culham et al., 1998; Alnaes et al., 2015; Dorum et al., 2016), which is involved in the functions of attentional transformation and reorientation, spatial attention, spatial working memory, and visual motion (Corbetta and Shulman, 2002; Ptak, 2012). Thus, a decrease in Microstate D parameters after exhaustive exercise may reduce the ability of the dorsal attention network to interact with information by decreasing the efficiency of this network, which in turn may lead to a decrease in athlete-related cognition or motor performance and affect competitive performance.

Finally, there was a significant increase in transition probability between microstate A associated with the language processing network and microstate C, associated with the salience network, after exhaustion motion (Seeley et al., 2007; Britz et al., 2010). There were also significantly lower transition probabilities between Microstate B, associated with the visual network and Microstate D, associated with the attention network (Britz et al., 2010). Spring et al. (2018) found a significantly higher transition from Microstate A to Microstate C after exhaustive cycling, which is consistent with the study. However, they did not find a change in the transition probability from Microstate C to Microstate A and Microstate B to D, which may be due to differences in exercise patterns or intensities. It has been shown that transitions between microstates may represent the transitions or sequential activation of different neural networks (Khanna et al., 2015). In the study of neurological disorders, changes in microstates are often considered activity or connectivity anomalies corresponding to functional networks, explaining associated cognitive or behavioral deficits (Bochet et al., 2021; Wang et al., 2021). Thus, acute motor fatigue may lead to increased transitions between the speech processing network and the convexity network and decreased transitions between the visual and attention networks.

### 4.2. Large-scale brain network energy analysis

In this study, we found that the energy of high-frequency alpha and low-frequency beta bands showed an increasing trend on all eight large-scale brain networks after exhaustive exercise, but no significant differences were found. This may be because, after exhaustive exercise, high-frequency alpha and low-frequency beta wave energy is increased over the whole brain rather than specific regions (Crabbe and Dishman, 2004; Bailey et al., 2008). Notably, the energy of the high-frequency beta band over the visual network was significantly higher after exhaustive exercise. The visual network belongs to the primary perceptual network, a posterior neural network that includes the occipital cortex and temporo-occipital regions of the retina and is dedicated to the information processing of visually relevant stimuli (Mantini et al., 2007). Beta waves are the common high-frequency rhythm that people experience while awake. In healthy adults, beta rhythms are mainly located in the frontal, parietal, temporal and occipital regions (Mantini et al., 2007). they appear when adults are in states of alertness, are involved in decision-making, are making judgments, and are solving problems. Cognitive activities such as attention, anticipation, and decision-making are inseparable from the participation of visual networks, and efficient visual search behaviors are crucial to athletes’ perceptual-cognitive abilities during the competition (Mann et al., 2007).

Studies have shown that athletes’ cognitive or behavioral performance decreases with fatigue during prolonged, high-intensity competition. Alder et al. (2019) found that high physiological load decreases visual search efficiency and prediction accuracy in badminton players. Casanova et al. (2013) found that prolonged intermittent exercise causes a decrease in the efficiency of specialized visual search in soccer players. Smith et al. (2016) found that mental fatigue led to a decrease in soccer players’ specific visual anticipation, and Alder et al. (2021) also found that either exercise or mental fatigue alone led to a decrease in soccer players’ specific visual anticipation, which was further worsened when the two were combined. Bullock and Giesbrecht (2014) found that strenuous exercise similarly decreased general visual search efficiency in non-athletes. Thus, high energy in the high-frequency beta band on the visual network after exhaustive exercise may not represent an increase in cognitive or behavioral performance associated with visual information processing but is more likely a decrease. The study found that superior athletes had lower neural activity in specific brain regions but better task performance when completing relevant cognitive or motor tasks than novices (Del Percio et al., 2009; Babiloni et al., 2010; Guo et al., 2017). Researchers have used the “neural efficiency” hypothesis to explain this phenomenon, suggesting that good athletes, after long training, can perform tasks well in an automated, less neurologically intensive processing mode, such that specific brain activity levels are lower but are a sign of high neural efficiency (Callan and Naito, 2014). This may also imply that when people perform the same cognitive task in poor physical condition, a higher level of brain activity is a sign of reduced neural efficiency, as more cognitive resources are used to maintain task performance. Therefore, the high energy in the high-frequency beta band on the visual network after exhaustive exercise might be caused by participants maintaining visually relevant cognitive performance in a controlled, more effortful processing mode that requires control, suggesting a decrease in neural efficiency that may explain previous claims of decreased visually relevant cognitive performance due to high-intensity or exhaustive exercise.

Therefore, it is feasible to monitor the effects of acute exercise fatigue on the neural activity of the body using EEG microstates and resting-state cortical rhythm techniques. The effect of exercise modality, intensity, duration or frequency on the energy of EEG rhythms on micro-state or large-scale brain networks could be further explored in future studies, as this could be informative for the development of training programs for athletes and exercise prescriptions for people who want to improve relevant cognitive or behavioral performance through exercise.

## 5. Limitations

First, our study did not measure the relevant cognitive or behavioral performance of the participants before and after exhaustive exercise. Therefore, the above conclusion that changes in microstate parameters and EEG rhythm energy in large-scale brain networks affect certain cognitive or behavioral performances should be further explored in future studies. Second, the reliability of the comparison of results between similar studies remains to be explored due to differences in exercise patterns (Brummer et al., 2011), the participants’ exercise preferences (Schneider et al., 2009) and physical activity levels, EEG models, and analysis methods. Third, our study measured EEG only in the immediate post-exertional period of the participants and therefore could not assess the characteristics of their brain activity during recovery (Gutmann et al., 2018; Spring et al., 2018), which is nevertheless informative for the development of training programs for athletes. Fourth, compared to 64, 128, or 256 electrodes, the accuracy of assigning the cortical source space network by 32 electrodes is lower in this study and should be further improved in future studies.

## 6. Conclusion

In this study, the effects of acute motor fatigue on large-scale brain functional networks were investigated for the first time by EEG micro-state and resting-state cortical rhythm techniques. The present study showed that exhaustive exercise could influence the activity of higher cognitive processing networks (Microstate C and D). At the same time, it had less effect on the activity of primary perceptual networks (Microstate A and B). In addition, the high energy in the high-frequency beta band on the participants’ visual network after exhaustive exercise might indicate decreased neural efficiency.

## Data availability statement

The raw data supporting the conclusions of this article will be made available by the authors, without undue reservation.

## Ethics statement

The studies involving human participants were reviewed and approved by the Academic Ethics Committee of Shaanxi Normal University. The patients/participants provided their written informed consent to participate in this study.

## Author contributions

SZ and HL: writing—original draft preparation. YG and AC: supervision. AC: funding acquisition. All authors have read and agreed to the published version of the manuscript.

## Footnotes

1
http://www.leixulab.net/software.asp

2
http://fieldtrip.fcdonders.nl/download.php

3
https://sites.google.com/site/cartoolcommunity/
